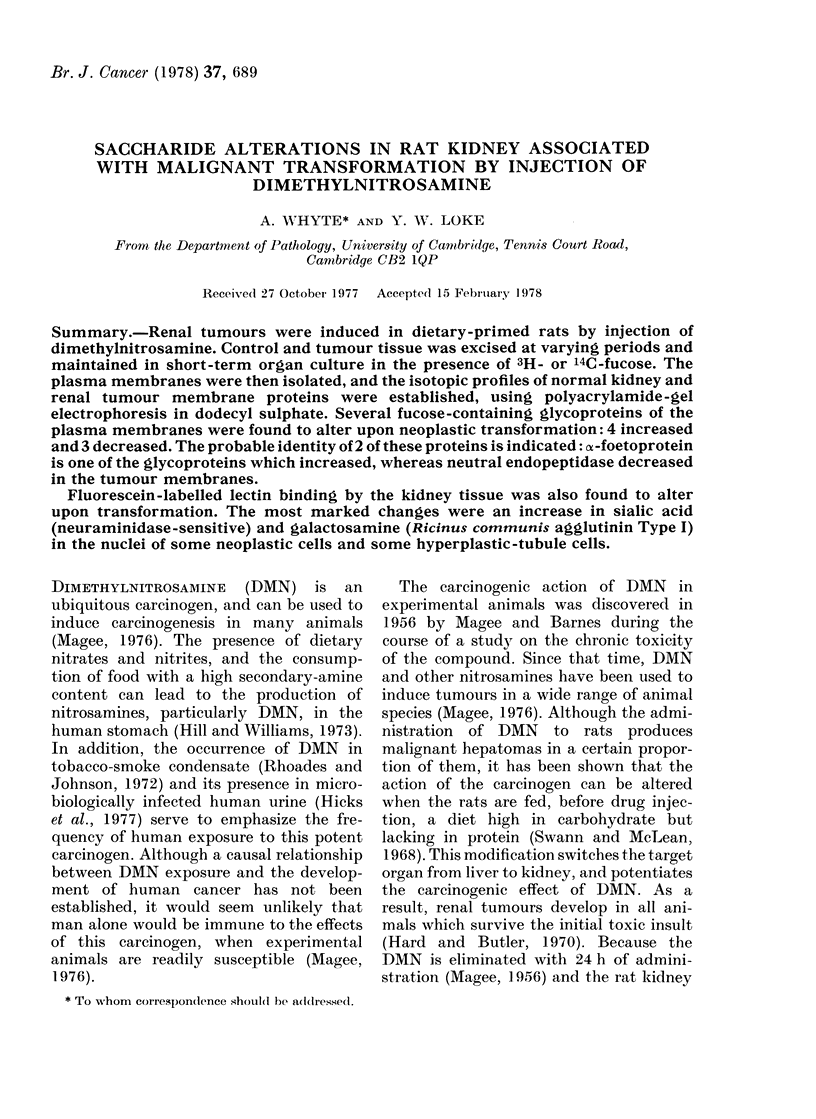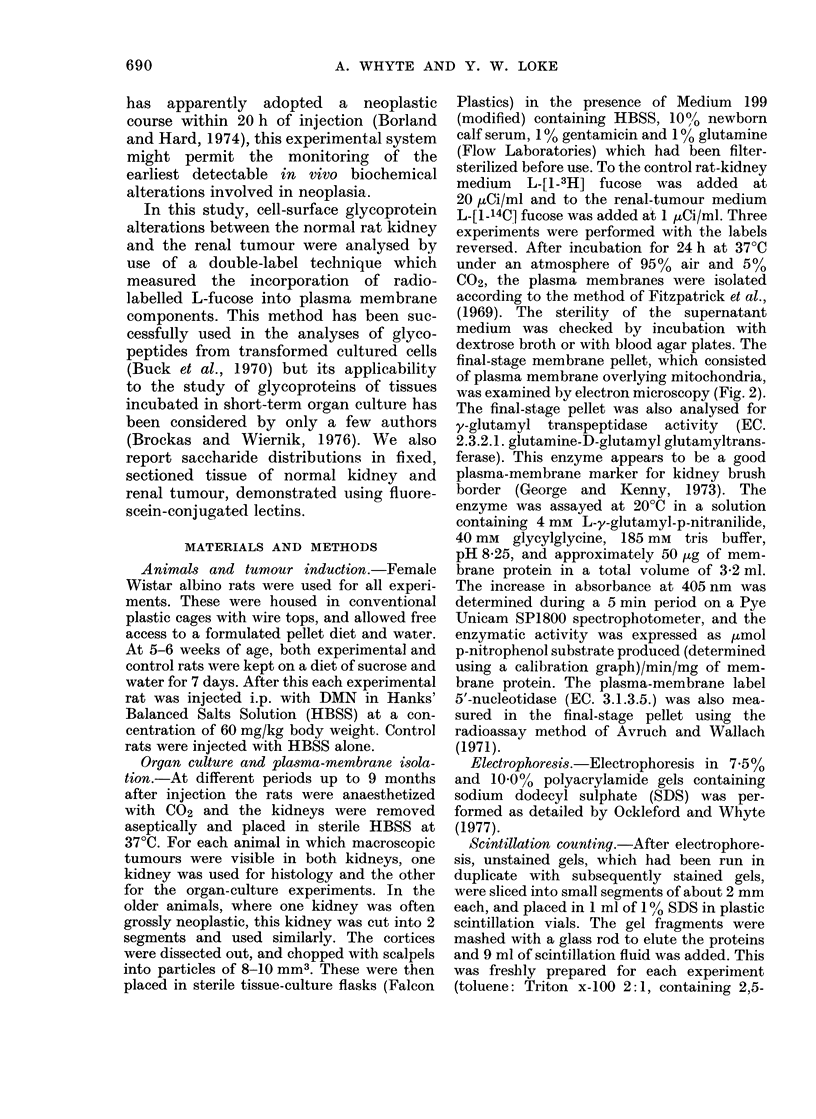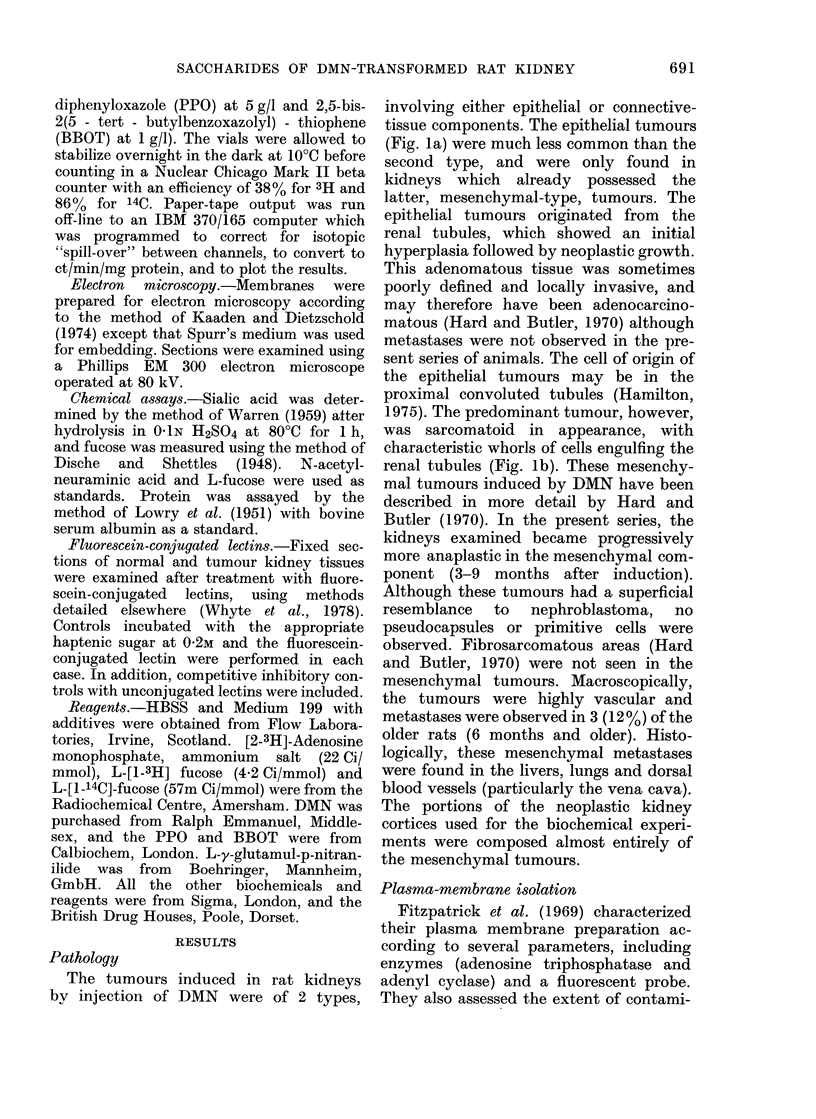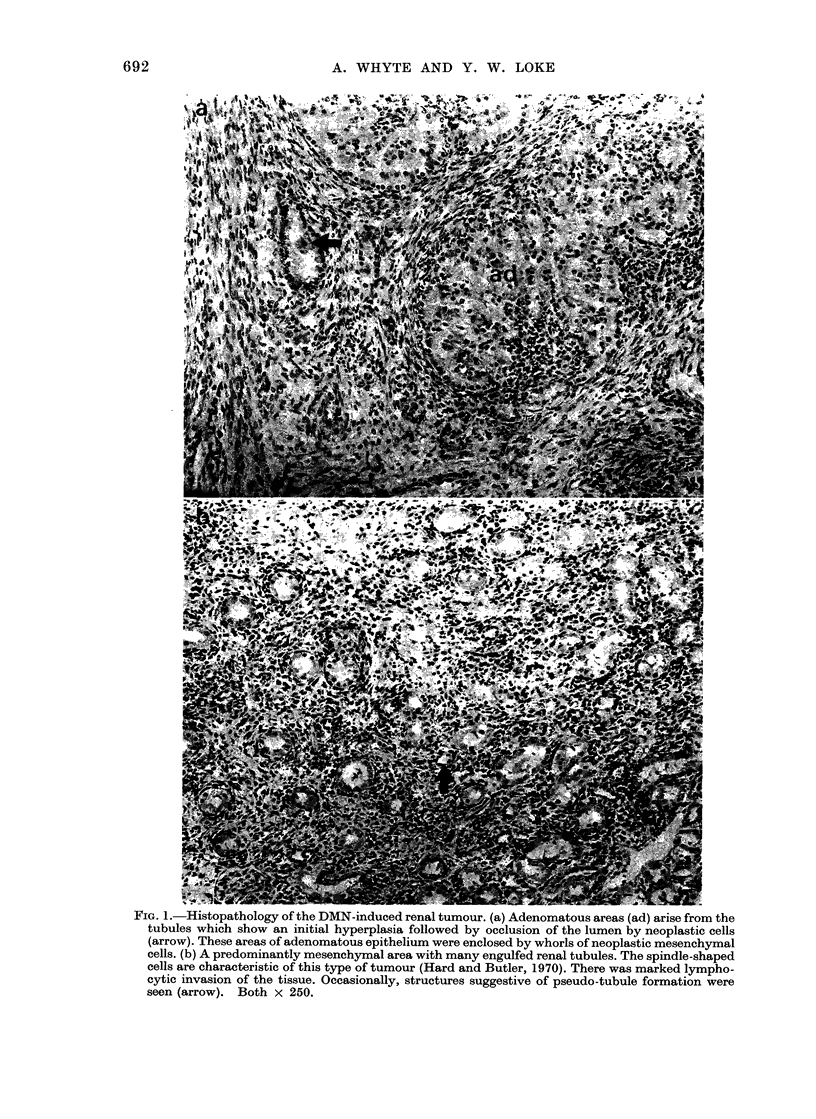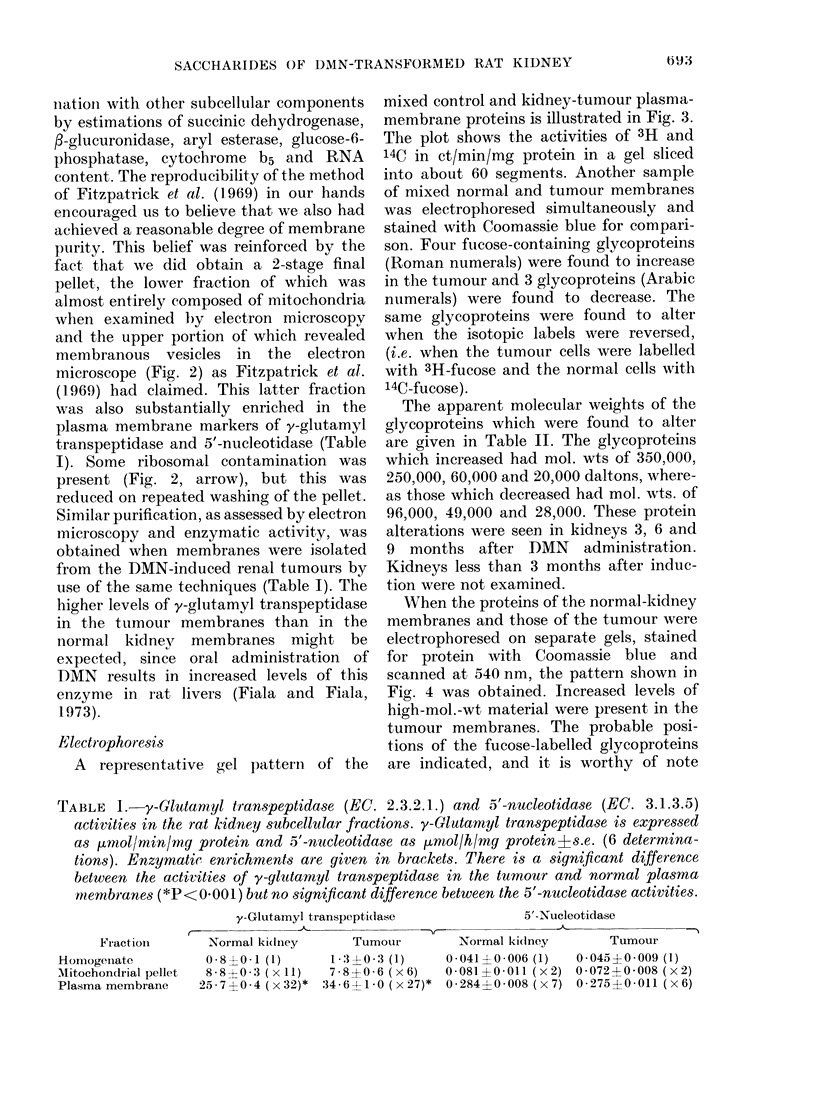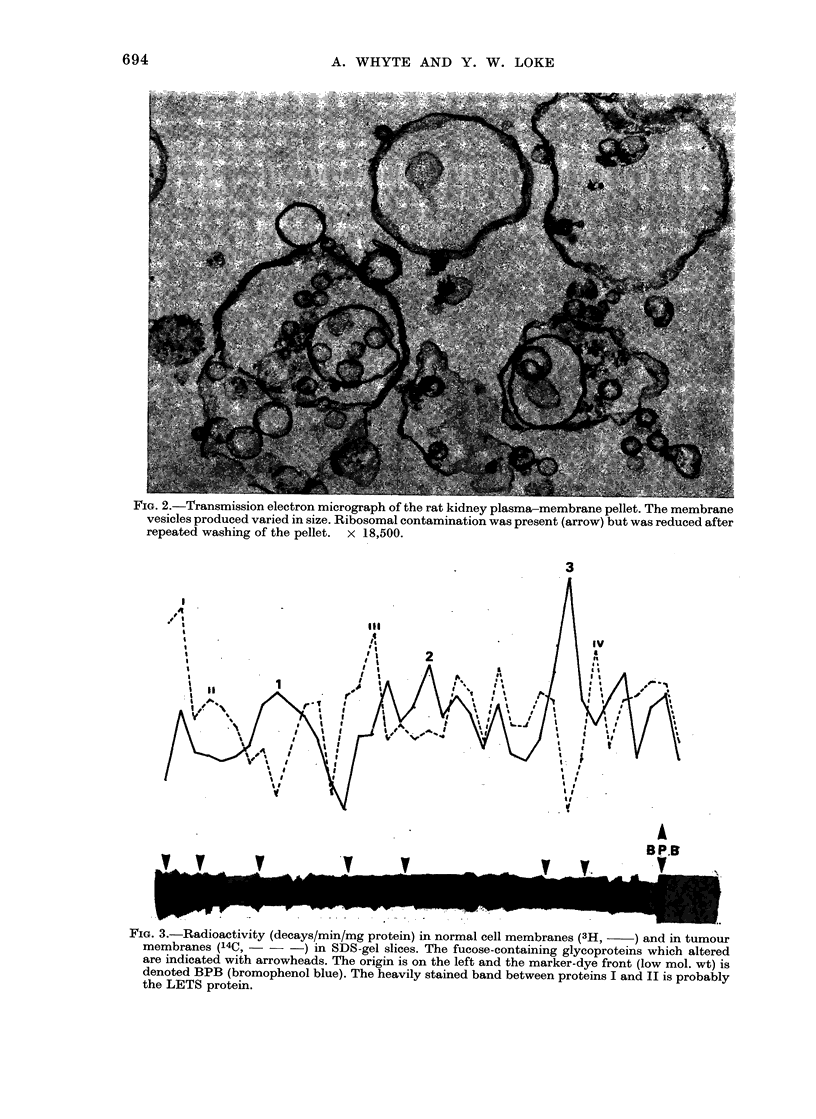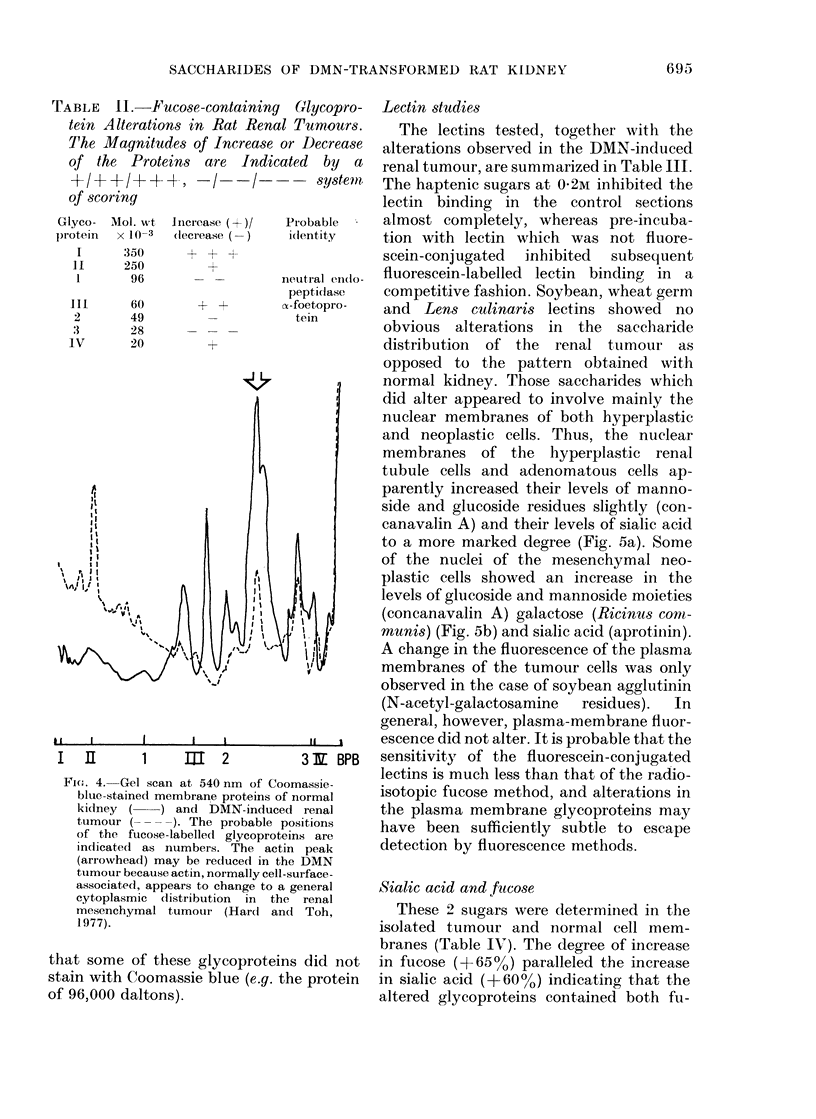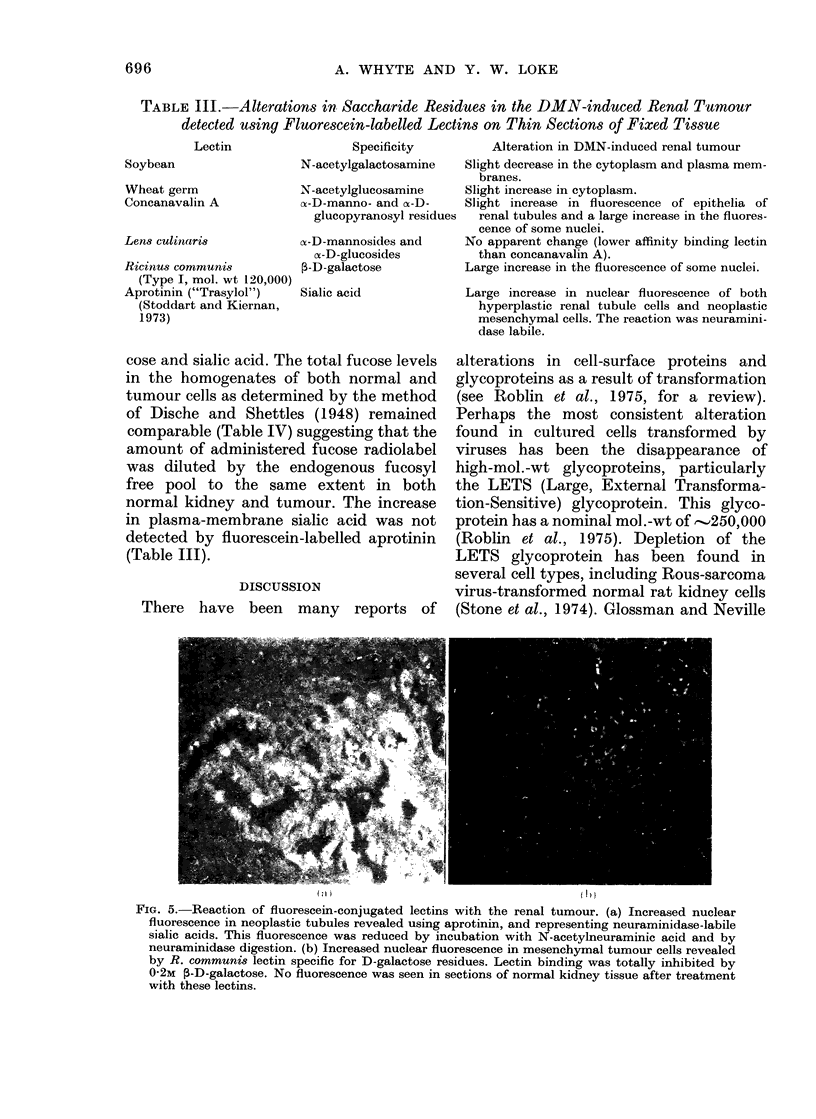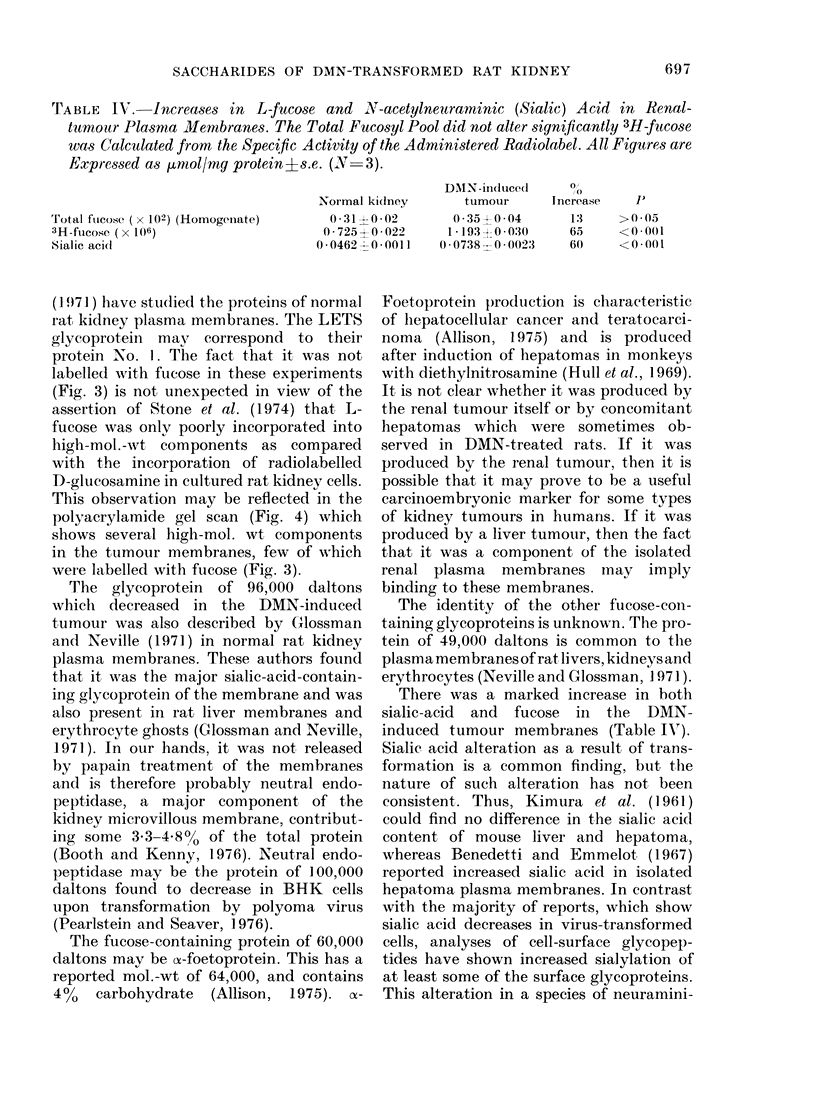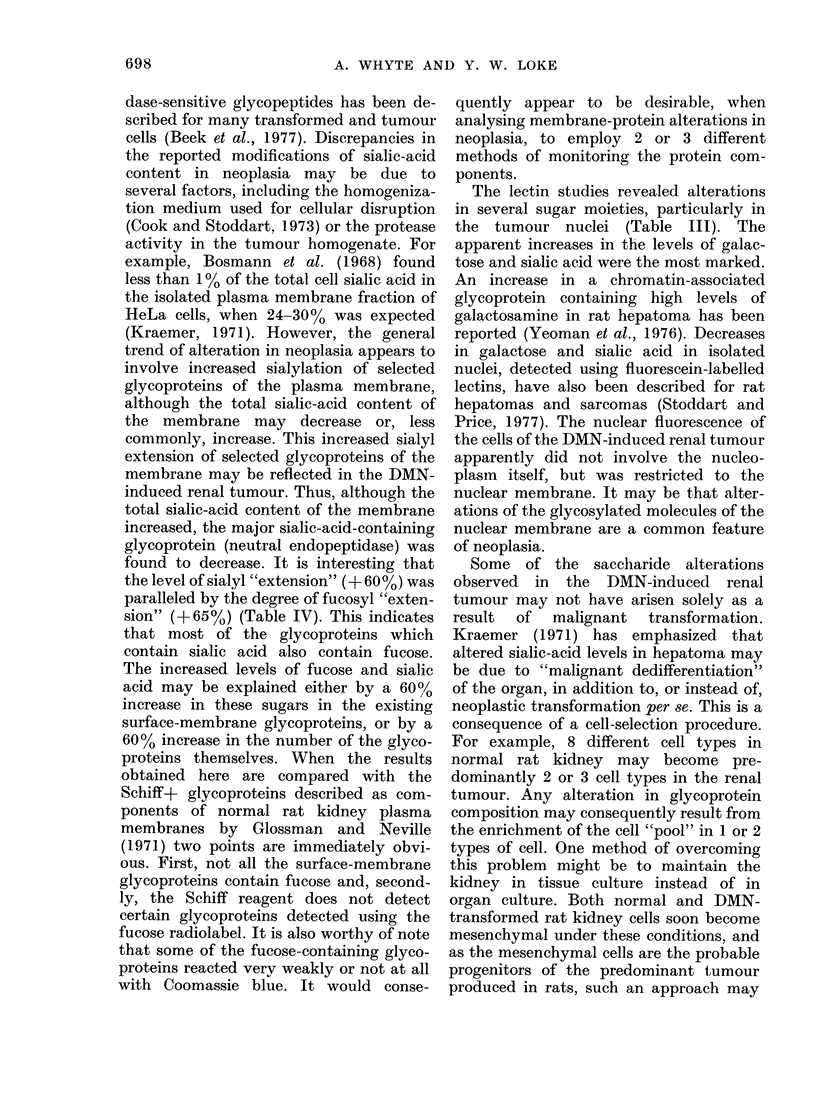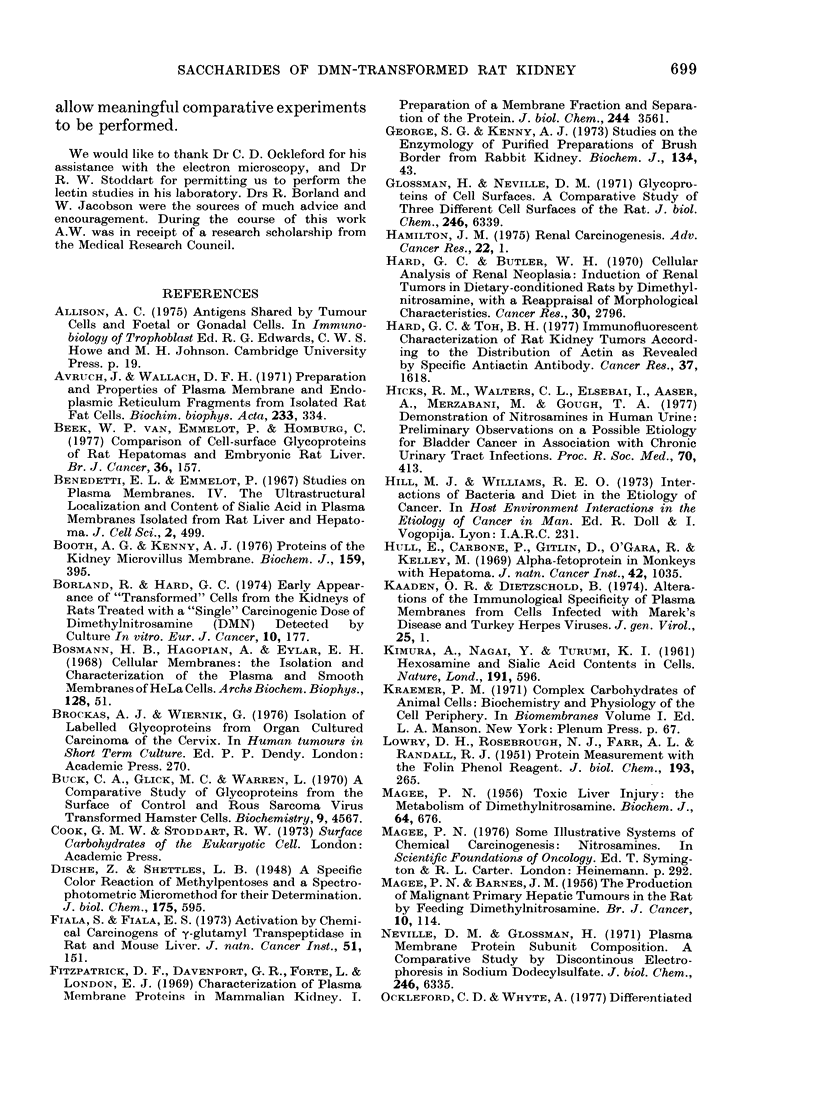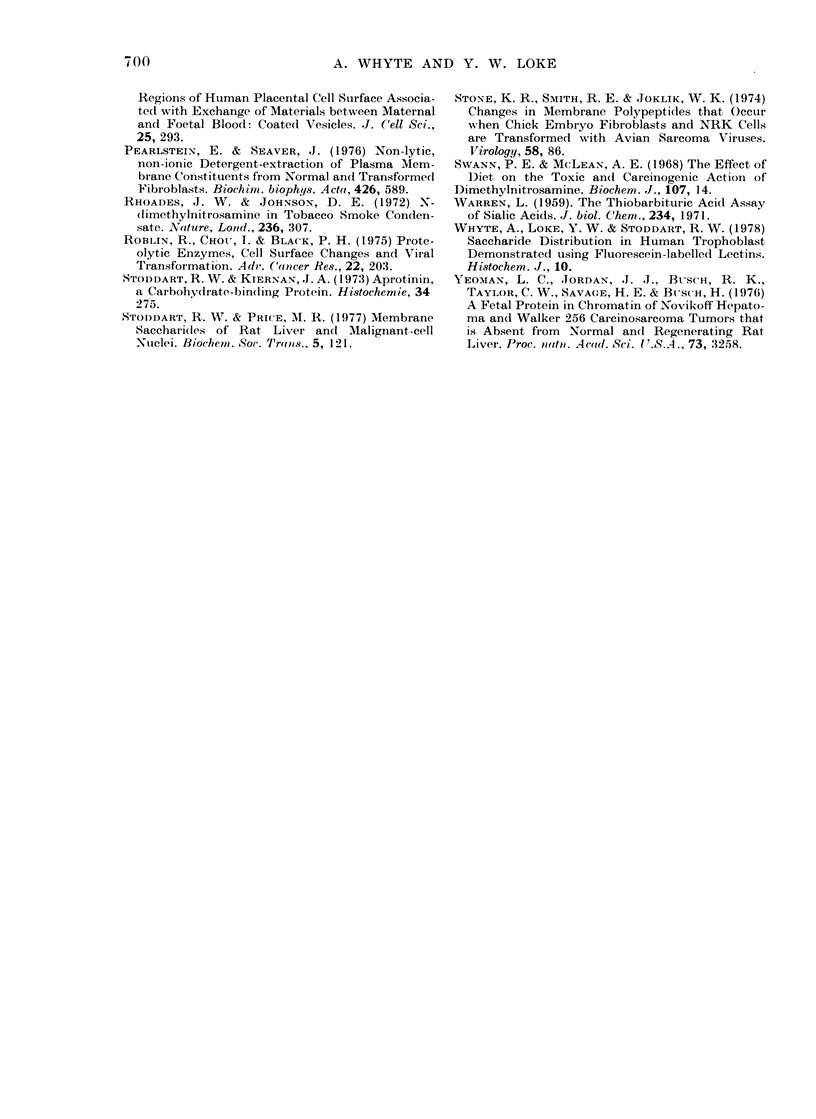# Saccharide alterations in rat kidney associated with malignant transformation by injection of dimethylnitrosamine.

**DOI:** 10.1038/bjc.1978.106

**Published:** 1978-05

**Authors:** A. Whyte, Y. W. Loke

## Abstract

**Images:**


*Br. J. Cancer* (1978) **37**, 689

### SACCHARIDE ALTERATIONS IN RAT KIDNEY ASSOCIATED WITH MALIGNANT TRANSFORMATION BY INJECTION OF DIMETHYLNITROSAMINE

A. WHYTE* AND Y. W. LOKE

*From the Department of Pathology, University of Cambridge, Tennis Court Road, Cambridge CB2 1QP*

Received 27 October 1977 Accepted 15 February 1978

**Summary.—Renal tumours were induced in dietary-primed rats by injection of dimethylnitrosamine. Control and tumour tissue was excised at varying periods and maintained in short-term organ culture in the presence of $^{3}H$- or $^{14}C$-fucose. The plasma membranes were then isolated, and the isotopic profiles of normal kidney and renal tumour membrane proteins were established, using polyacrylamide-gel electrophoresis in dodecyl sulphate. Several fucose-containing glycoproteins of the plasma membranes were found to alter upon neoplastic transformation: 4 increased and 3 decreased. The probable identity of 2 of these proteins is indicated: $\alpha$-foetoprotein is one of the glycoproteins which increased, whereas neutral endopeptidase decreased in the tumour membranes.**

**Fluorescein-labelled lectin binding by the kidney tissue was also found to alter upon transformation. The most marked changes were an increase in sialic acid (neuraminidase-sensitive) and galactosamine (*Ricinus communis* agglutinin Type I) in the nuclei of some neoplastic cells and some hyperplastic-tubule cells.**

DIMETHYLNITROSAMINE (DMN) is an ubiquitous carcinogen, and can be used to induce carcinogenesis in many animals (Magee, 1976). The presence of dietary nitrates and nitrites, and the consumption of food with a high secondary-amine content can lead to the production of nitrosamines, particularly DMN, in the human stomach (Hill and Williams, 1973). In addition, the occurrence of DMN in tobacco-smoke condensate (Rhoades and Johnson, 1972) and its presence in microbiologically infected human urine (Hicks *et al.*, 1977) serve to emphasize the frequency of human exposure to this potent carcinogen. Although a causal relationship between DMN exposure and the development of human cancer has not been established, it would seem unlikely that man alone would be immune to the effects of this carcinogen, when experimental animals are readily susceptible (Magee, 1976).

The carcinogenic action of DMN in experimental animals was discovered in 1956 by Magee and Barnes during the course of a study on the chronic toxicity of the compound. Since that time, DMN and other nitrosamines have been used to induce tumours in a wide range of animal species (Magee, 1976). Although the administration of DMN to rats produces malignant hepatomas in a certain proportion of them, it has been shown that the action of the carcinogen can be altered when the rats are fed, before drug injection, a diet high in carbohydrate but lacking in protein (Swann and McLean, 1968). This modification switches the target organ from liver to kidney, and potentiates the carcinogenic effect of DMN. As a result, renal tumours develop in all animals which survive the initial toxic insult (Hard and Butler, 1970). Because the DMN is eliminated with 24 h of administration (Magee, 1956) and the rat kidney

* To whom correspondence should be addressed.

has apparently adopted a neoplastic course within 20 h of injection (Borland and Hard, 1974), this experimental system might permit the monitoring of the earliest detectable *in vivo* biochemical alterations involved in neoplasia.

In this study, cell-surface glycoprotein alterations between the normal rat kidney and the renal tumour were analysed by use of a double-label technique which measured the incorporation of radio-labelled L-fucose into plasma membrane components. This method has been successfully used in the analyses of glycopeptides from transformed cultured cells (Buck *et al.*, 1970) but its applicability to the study of glycoproteins of tissues incubated in short-term organ culture has been considered by only a few authors (Brockas and Wiernik, 1976). We also report saccharide distributions in fixed, sectioned tissue of normal kidney and renal tumour, demonstrated using fluorescein-conjugated lectins.

### MATERIALS AND METHODS

*Animals and tumour induction.*—Female Wistar albino rats were used for all experiments. These were housed in conventional plastic cages with wire tops, and allowed free access to a formulated pellet diet and water. At 5–6 weeks of age, both experimental and control rats were kept on a diet of sucrose and water for 7 days. After this each experimental rat was injected i.p. with DMN in Hanks' Balanced Salts Solution (HBSS) at a concentration of 60 mg/kg body weight. Control rats were injected with HBSS alone.

*Organ culture and plasma-membrane isolation.*—At different periods up to 9 months after injection the rats were anaesthetized with $CO_2$ and the kidneys were removed aseptically and placed in sterile HBSS at 37°C. For each animal in which macroscopic tumours were visible in both kidneys, one kidney was used for histology and the other for the organ-culture experiments. In the older animals, where one kidney was often grossly neoplastic, this kidney was cut into 2 segments and used similarly. The cortices were dissected out, and chopped with scalpels into particles of 8–10 $mm^3$. These were then placed in sterile tissue-culture flasks (Falcon Plastics) in the presence of Medium 199 (modified) containing HBSS, 10% newborn calf serum, 1% gentamicin and 1% glutamine (Flow Laboratories) which had been filter-sterilized before use. To the control rat-kidney medium L-[1-$^3$H] fucose was added at 20 $\mu$Ci/ml and to the renal-tumour medium L-[1-$^{14}$C] fucose was added at 1 $\mu$Ci/ml. Three experiments were performed with the labels reversed. After incubation for 24 h at 37°C under an atmosphere of 95% air and 5% $CO_2$, the plasma membranes were isolated according to the method of Fitzpatrick *et al.*, (1969). The sterility of the supernatant medium was checked by incubation with dextrose broth or with blood agar plates. The final-stage membrane pellet, which consisted of plasma membrane overlying mitochondria, was examined by electron microscopy (Fig. 2). The final-stage pellet was also analysed for $\gamma$-glutamyl transpeptidase activity (EC. 2.3.2.1. glutamine-D-glutamyl glutamyltransferase). This enzyme appears to be a good plasma-membrane marker for kidney brush border (George and Kenny, 1973). The enzyme was assayed at 20°C in a solution containing 4 mM L-$\gamma$-glutamyl-p-nitranilide, 40 mM glycylglycine, 185 mM tris buffer, pH 8·25, and approximately 50 $\mu$g of membrane protein in a total volume of 3·2 ml. The increase in absorbance at 405 nm was determined during a 5 min period on a Pye Unicam SP1800 spectrophotometer, and the enzymatic activity was expressed as $\mu$mol p-nitrophenol substrate produced (determined using a calibration graph)/min/mg of membrane protein. The plasma-membrane label 5′-nucleotidase (EC. 3.1.3.5.) was also measured in the final-stage pellet using the radioassay method of Avruch and Wallach (1971).

*Electrophoresis.*—Electrophoresis in 7·5% and 10·0% polyacrylamide gels containing sodium dodecyl sulphate (SDS) was performed as detailed by Ockleford and Whyte (1977).

*Scintillation counting.*—After electrophoresis, unstained gels, which had been run in duplicate with subsequently stained gels, were sliced into small segments of about 2 mm each, and placed in 1 ml of 1% SDS in plastic scintillation vials. The gel fragments were mashed with a glass rod to elute the proteins and 9 ml of scintillation fluid was added. This was freshly prepared for each experiment (toluene: Triton x-100 2:1, containing 2,5-

diphenyloxazole (PPO) at 5 g/l and 2,5-bis-2(5 - tert - butylbenzoxazolyl) - thiophene (BBOT) at 1 g/l). The vials were allowed to stabilize overnight in the dark at 10°C before counting in a Nuclear Chicago Mark II beta counter with an efficiency of 38% for $^3H$ and 86% for $^{14}C$. Paper-tape output was run off-line to an IBM 370/165 computer which was programmed to correct for isotopic "spill-over" between channels, to convert to ct/min/mg protein, and to plot the results.

*Electron microscopy.*—Membranes were prepared for electron microscopy according to the method of Kaaden and Dietzschold (1974) except that Spurr's medium was used for embedding. Sections were examined using a Phillips EM 300 electron microscope operated at 80 kV.

*Chemical assays.*—Sialic acid was determined by the method of Warren (1959) after hydrolysis in 0·1N $H_2SO_4$ at 80°C for 1 h, and fucose was measured using the method of Dische and Shettles (1948). N-acetyl-neuraminic acid and L-fucose were used as standards. Protein was assayed by the method of Lowry *et al.* (1951) with bovine serum albumin as a standard.

*Fluorescein-conjugated lectins.*—Fixed sections of normal and tumour kidney tissues were examined after treatment with fluorescein-conjugated lectins, using methods detailed elsewhere (Whyte *et al.*, 1978). Controls incubated with the appropriate haptenic sugar at 0·2M and the fluorescein-conjugated lectin were performed in each case. In addition, competitive inhibitory controls with unconjugated lectins were included.

*Reagents.*—HBSS and Medium 199 with additives were obtained from Flow Laboratories, Irvine, Scotland. [2-$^3$H]-Adenosine monophosphate, ammonium salt (22 Ci/mmol), L-[1-$^3$H] fucose (4·2 Ci/mmol) and L-[1-$^{14}$C]-fucose (57m Ci/mmol) were from the Radiochemical Centre, Amersham. DMN was purchased from Ralph Emmanuel, Middlesex, and the PPO and BBOT were from Calbiochem, London. L-γ-glutamul-p-nitranilide was from Boehringer, Mannheim, GmbH. All the other biochemicals and reagents were from Sigma, London, and the British Drug Houses, Poole, Dorset.

### RESULTS

### *Pathology*

The tumours induced in rat kidneys by injection of DMN were of 2 types, involving either epithelial or connective-tissue components. The epithelial tumours (Fig. 1a) were much less common than the second type, and were only found in kidneys which already possessed the latter, mesenchymal-type, tumours. The epithelial tumours originated from the renal tubules, which showed an initial hyperplasia followed by neoplastic growth. This adenomatous tissue was sometimes poorly defined and locally invasive, and may therefore have been adenocarcinomatous (Hard and Butler, 1970) although metastases were not observed in the present series of animals. The cell of origin of the epithelial tumours may be in the proximal convoluted tubules (Hamilton, 1975). The predominant tumour, however, was sarcomatoid in appearance, with characteristic whorls of cells engulfing the renal tubules (Fig. 1b). These mesenchymal tumours induced by DMN have been described in more detail by Hard and Butler (1970). In the present series, the kidneys examined became progressively more anaplastic in the mesenchymal component (3–9 months after induction). Although these tumours had a superficial resemblance to nephroblastoma, no pseudocapsules or primitive cells were observed. Fibrosarcomatous areas (Hard and Butler, 1970) were not seen in the mesenchymal tumours. Macroscopically, the tumours were highly vascular and metastases were observed in 3 (12%) of the older rats (6 months and older). Histologically, these mesenchymal metastases were found in the livers, lungs and dorsal blood vessels (particularly the vena cava). The portions of the neoplastic kidney cortices used for the biochemical experiments were composed almost entirely of the mesenchymal tumours.

### *Plasma-membrane isolation*

Fitzpatrick *et al.* (1969) characterized their plasma membrane preparation according to several parameters, including enzymes (adenosine triphosphatase and adenyl cyclase) and a fluorescent probe. They also assessed the extent of contami-

FIG. 1.—Histopathology of the DMN-induced renal tumour. (a) Adenomatous areas (ad) arise from the tubules which show an initial hyperplasia followed by occlusion of the lumen by neoplastic cells (arrow). These areas of adenomatous epithelium were enclosed by whorls of neoplastic mesenchymal cells. (b) A predominantly mesenchymal area with many engulfed renal tubules. The spindle-shaped cells are characteristic of this type of tumour (Hard and Butler, 1970). There was marked lymphocytic invasion of the tissue. Occasionally, structures suggestive of pseudo-tubule formation were seen (arrow). Both × 250.

nation with other subcellular components by estimations of succinic dehydrogenase, β-glucuronidase, aryl esterase, glucose-6-phosphatase, cytochrome $b_5$ and RNA content. The reproducibility of the method of Fitzpatrick *et al.* (1969) in our hands encouraged us to believe that we also had achieved a reasonable degree of membrane purity. This belief was reinforced by the fact that we did obtain a 2-stage final pellet, the lower fraction of which was almost entirely composed of mitochondria when examined by electron microscopy and the upper portion of which revealed membranous vesicles in the electron microscope (Fig. 2) as Fitzpatrick *et al.* (1969) had claimed. This latter fraction was also substantially enriched in the plasma membrane markers of γ-glutamyl transpeptidase and 5′-nucleotidase (Table I). Some ribosomal contamination was present (Fig. 2, arrow), but this was reduced on repeated washing of the pellet. Similar purification, as assessed by electron microscopy and enzymatic activity, was obtained when membranes were isolated from the DMN-induced renal tumours by use of the same techniques (Table I). The higher levels of γ-glutamyl transpeptidase in the tumour membranes than in the normal kidney membranes might be expected, since oral administration of DMN results in increased levels of this enzyme in rat livers (Fiala and Fiala, 1973).

*Electrophoresis*

A representative gel pattern of the mixed control and kidney-tumour plasma-membrane proteins is illustrated in Fig. 3. The plot shows the activities of $^{3}H$ and $^{14}C$ in ct/min/mg protein in a gel sliced into about 60 segments. Another sample of mixed normal and tumour membranes was electrophoresed simultaneously and stained with Coomassie blue for comparison. Four fucose-containing glycoproteins (Roman numerals) were found to increase in the tumour and 3 glycoproteins (Arabic numerals) were found to decrease. The same glycoproteins were found to alter when the isotopic labels were reversed, (*i.e.* when the tumour cells were labelled with $^{3}H$-fucose and the normal cells with $^{14}C$-fucose).

The apparent molecular weights of the glycoproteins which were found to alter are given in Table II. The glycoproteins which increased had mol. wts of 350,000, 250,000, 60,000 and 20,000 daltons, whereas those which decreased had mol. wts. of 96,000, 49,000 and 28,000. These protein alterations were seen in kidneys 3, 6 and 9 months after DMN administration. Kidneys less than 3 months after induction were not examined.

When the proteins of the normal-kidney membranes and those of the tumour were electrophoresed on separate gels, stained for protein with Coomassie blue and scanned at 540 nm, the pattern shown in Fig. 4 was obtained. Increased levels of high-mol.-wt material were present in the tumour membranes. The probable positions of the fucose-labelled glycoproteins are indicated, and it is worthy of note

TABLE I.—*γ-Glutamyl transpeptidase (EC. 2.3.2.1.) and 5′-nucleotidase (EC. 3.1.3.5) activities in the rat kidney subcellular fractions. γ-Glutamyl transpeptidase is expressed as μmol/min/mg protein and 5′-nucleotidase as μmol/h/mg protein±s.e. (6 determinations). Enzymatic enrichments are given in brackets. There is a significant difference between the activities of γ-glutamyl transpeptidase in the tumour and normal plasma membranes* (*P<0·001) *but no significant difference between the 5′-nucleotidase activities.*

| | γ-Glutamyl transpeptidase | | 5′-Nucleotidase | |
|---|---|---|---|---|
| Fraction | Normal kidney | Tumour | Normal kidney | Tumour |
| Homogenate | 0·8±0·1 (1) | 1·3±0·3 (1) | 0·041±0·006 (1) | 0·045±0·009 (1) |
| Mitochondrial pellet | 8·8±0·3 (×11) | 7·8±0·6 (×6) | 0·081±0·011 (×2) | 0·072±0·008 (×2) |
| Plasma membrane | 25·7±0·4 (×32)* | 34·6±1·0 (×27)* | 0·284±0·008 (×7) | 0·275±0·011 (×6) |

FIG. 2.—Transmission electron micrograph of the rat kidney plasma–membrane pellet. The membrane vesicles produced varied in size. Ribosomal contamination was present (arrow) but was reduced after repeated washing of the pellet. × 18,500.

FIG. 3.—Radioactivity (decays/min/mg protein) in normal cell membranes ($^3H$, ——) and in tumour membranes ($^{14}C$, — — —) in SDS-gel slices. The fucose-containing glycoproteins which altered are indicated with arrowheads. The origin is on the left and the marker-dye front (low mol. wt) is denoted BPB (bromophenol blue). The heavily stained band between proteins I and II is probably the LETS protein.

TABLE II.—*Fucose-containing Glycoprotein Alterations in Rat Renal Tumours. The Magnitudes of Increase or Decrease of the Proteins are Indicated by a +/++/+++, –/– –/– – – system of scoring*

| Glycoprotein | Mol. wt × $10^{-3}$ | Increase (+)/ decrease (–) | Probable identity |
|---|---|---|---|
| I | 350 | + + + | |
| II | 250 | + | |
| 1 | 96 | – – | neutral endopeptidase |
| III | 60 | + + | α-foetoprotein |
| 2 | 49 | – | |
| 3 | 28 | – – – | |
| IV | 20 | + | |

FIG. 4.—Gel scan at 540 nm of Coomassie-blue-stained membrane proteins of normal kidney (——) and DMN-induced renal tumour (– – – –). The probable positions of the fucose-labelled glycoproteins are indicated as numbers. The actin peak (arrowhead) may be reduced in the DMN tumour because actin, normally cell-surface-associated, appears to change to a general cytoplasmic distribution in the renal mesenchymal tumour (Hard and Toh, 1977).

that some of these glycoproteins did not stain with Coomassie blue (*e.g.* the protein of 96,000 daltons).

*Lectin studies*

The lectins tested, together with the alterations observed in the DMN-induced renal tumour, are summarized in Table III. The haptenic sugars at 0·2M inhibited the lectin binding in the control sections almost completely, whereas pre-incubation with lectin which was not fluorescein-conjugated inhibited subsequent fluorescein-labelled lectin binding in a competitive fashion. Soybean, wheat germ and *Lens culinaris* lectins showed no obvious alterations in the saccharide distribution of the renal tumour as opposed to the pattern obtained with normal kidney. Those saccharides which did alter appeared to involve mainly the nuclear membranes of both hyperplastic and neoplastic cells. Thus, the nuclear membranes of the hyperplastic renal tubule cells and adenomatous cells apparently increased their levels of mannoside and glucoside residues slightly (concanavalin A) and their levels of sialic acid to a more marked degree (Fig. 5a). Some of the nuclei of the mesenchymal neoplastic cells showed an increase in the levels of glucoside and mannoside moieties (concanavalin A) galactose (*Ricinus communis*) (Fig. 5b) and sialic acid (aprotinin). A change in the fluorescence of the plasma membranes of the tumour cells was only observed in the case of soybean agglutinin (N-acetyl-galactosamine residues). In general, however, plasma-membrane fluorescence did not alter. It is probable that the sensitivity of the fluorescein-conjugated lectins is much less than that of the radioisotopic fucose method, and alterations in the plasma membrane glycoproteins may have been sufficiently subtle to escape detection by fluorescence methods.

*Sialic acid and fucose*

These 2 sugars were determined in the isolated tumour and normal cell membranes (Table IV). The degree of increase in fucose (+65%) paralleled the increase in sialic acid (+60%) indicating that the altered glycoproteins contained both fu-

TABLE III.—*Alterations in Saccharide Residues in the DMN-induced Renal Tumour detected using Fluorescein-labelled Lectins on Thin Sections of Fixed Tissue*

| Lectin | Specificity | Alteration in DMN-induced renal tumour |
|---|---|---|
| Soybean | N-acetylgalactosamine | Slight decrease in the cytoplasm and plasma membranes. |
| Wheat germ | N-acetylglucosamine | Slight increase in cytoplasm. |
| Concanavalin A | α-D-manno- and α-D-glucopyranosyl residues | Slight increase in fluorescence of epithelia of renal tubules and a large increase in the fluorescence of some nuclei. |
| *Lens culinaris* | α-D-mannosides and α-D-glucosides | No apparent change (lower affinity binding lectin than concanavalin A). |
| *Ricinus communis* (Type I, mol. wt 120,000) | β-D-galactose | Large increase in the fluorescence of some nuclei. |
| Aprotinin ("Trasylol") (Stoddart and Kiernan, 1973) | Sialic acid | Large increase in nuclear fluorescence of both hyperplastic renal tubule cells and neoplastic mesenchymal cells. The reaction was neuraminidase labile. |

cose and sialic acid. The total fucose levels in the homogenates of both normal and tumour cells as determined by the method of Dische and Shettles (1948) remained comparable (Table IV) suggesting that the amount of administered fucose radiolabel was diluted by the endogenous fucosyl free pool to the same extent in both normal kidney and tumour. The increase in plasma-membrane sialic acid was not detected by fluorescein-labelled aprotinin (Table III).

### DISCUSSION

There have been many reports of alterations in cell-surface proteins and glycoproteins as a result of transformation (see Roblin *et al.*, 1975, for a review). Perhaps the most consistent alteration found in cultured cells transformed by viruses has been the disappearance of high-mol.-wt glycoproteins, particularly the LETS (Large, External Transformation-Sensitive) glycoprotein. This glycoprotein has a nominal mol.-wt of ~250,000 (Roblin *et al.*, 1975). Depletion of the LETS glycoprotein has been found in several cell types, including Rous-sarcoma virus-transformed normal rat kidney cells (Stone *et al.*, 1974). Glossman and Neville

(a) (b)

FIG. 5.—Reaction of fluorescein-conjugated lectins with the renal tumour. (a) Increased nuclear fluorescence in neoplastic tubules revealed using aprotinin, and representing neuraminidase-labile sialic acids. This fluorescence was reduced by incubation with N-acetylneuraminic acid and by neuraminidase digestion. (b) Increased nuclear fluorescence in mesenchymal tumour cells revealed by *R. communis* lectin specific for D-galactose residues. Lectin binding was totally inhibited by 0·2M β-D-galactose. No fluorescence was seen in sections of normal kidney tissue after treatment with these lectins.

TABLE IV.—*Increases in L-fucose and N-acetylneuraminic (Sialic) Acid in Renal-tumour Plasma Membranes. The Total Fucosyl Pool did not alter significantly $^3$H-fucose was Calculated from the Specific Activity of the Administered Radiolabel. All Figures are Expressed as μmol/mg protein±s.e. (N=3).*

| | Normal kidney | DMN-induced tumour | % Increase | $P$ |
|---|---|---|---|---|
| Total fucose ($\times 10^2$) (Homogenate) | 0·31±0·02 | 0·35±0·04 | 13 | >0·05 |
| $^3$H-fucose ($\times 10^6$) | 0·725±0·022 | 1·193±0·030 | 65 | <0·001 |
| Sialic acid | 0·0462±0·0011 | 0·0738±0·0023 | 60 | <0·001 |

(1971) have studied the proteins of normal rat kidney plasma membranes. The LETS glycoprotein may correspond to their protein No. 1. The fact that it was not labelled with fucose in these experiments (Fig. 3) is not unexpected in view of the assertion of Stone *et al.* (1974) that L-fucose was only poorly incorporated into high-mol.-wt components as compared with the incorporation of radiolabelled D-glucosamine in cultured rat kidney cells. This observation may be reflected in the polyacrylamide gel scan (Fig. 4) which shows several high-mol. wt components in the tumour membranes, few of which were labelled with fucose (Fig. 3).

The glycoprotein of 96,000 daltons which decreased in the DMN-induced tumour was also described by Glossman and Neville (1971) in normal rat kidney plasma membranes. These authors found that it was the major sialic-acid-containing glycoprotein of the membrane and was also present in rat liver membranes and erythrocyte ghosts (Glossman and Neville, 1971). In our hands, it was not released by papain treatment of the membranes and is therefore probably neutral endopeptidase, a major component of the kidney microvillous membrane, contributing some 3·3–4·8% of the total protein (Booth and Kenny, 1976). Neutral endopeptidase may be the protein of 100,000 daltons found to decrease in BHK cells upon transformation by polyoma virus (Pearlstein and Seaver, 1976).

The fucose-containing protein of 60,000 daltons may be α-foetoprotein. This has a reported mol.-wt of 64,000, and contains 4% carbohydrate (Allison, 1975). α-Foetoprotein production is characteristic of hepatocellular cancer and teratocarcinoma (Allison, 1975) and is produced after induction of hepatomas in monkeys with diethylnitrosamine (Hull *et al.*, 1969). It is not clear whether it was produced by the renal tumour itself or by concomitant hepatomas which were sometimes observed in DMN-treated rats. If it was produced by the renal tumour, then it is possible that it may prove to be a useful carcinoembryonic marker for some types of kidney tumours in humans. If it was produced by a liver tumour, then the fact that it was a component of the isolated renal plasma membranes may imply binding to these membranes.

The identity of the other fucose-containing glycoproteins is unknown. The protein of 49,000 daltons is common to the plasma membranes of rat livers, kidneys and erythrocytes (Neville and Glossman, 1971).

There was a marked increase in both sialic-acid and fucose in the DMN-induced tumour membranes (Table IV). Sialic acid alteration as a result of transformation is a common finding, but the nature of such alteration has not been consistent. Thus, Kimura *et al.* (1961) could find no difference in the sialic acid content of mouse liver and hepatoma, whereas Benedetti and Emmelot (1967) reported increased sialic acid in isolated hepatoma plasma membranes. In contrast with the majority of reports, which show sialic acid decreases in virus-transformed cells, analyses of cell-surface glycopeptides have shown increased sialylation of at least some of the surface glycoproteins. This alteration in a species of neuraminidase-sensitive glycopeptides has been described for many transformed and tumour cells (Beek *et al.*, 1977). Discrepancies in the reported modifications of sialic-acid content in neoplasia may be due to several factors, including the homogenization medium used for cellular disruption (Cook and Stoddart, 1973) or the protease activity in the tumour homogenate. For example, Bosmann *et al.* (1968) found less than 1% of the total cell sialic acid in the isolated plasma membrane fraction of HeLa cells, when 24–30% was expected (Kraemer, 1971). However, the general trend of alteration in neoplasia appears to involve increased sialylation of selected glycoproteins of the plasma membrane, although the total sialic-acid content of the membrane may decrease or, less commonly, increase. This increased sialyl extension of selected glycoproteins of the membrane may be reflected in the DMN-induced renal tumour. Thus, although the total sialic-acid content of the membrane increased, the major sialic-acid-containing glycoprotein (neutral endopeptidase) was found to decrease. It is interesting that the level of sialyl "extension" (+60%) was paralleled by the degree of fucosyl "extension" (+65%) (Table IV). This indicates that most of the glycoproteins which contain sialic acid also contain fucose. The increased levels of fucose and sialic acid may be explained either by a 60% increase in these sugars in the existing surface-membrane glycoproteins, or by a 60% increase in the number of the glycoproteins themselves. When the results obtained here are compared with the Schiff+ glycoproteins described as components of normal rat kidney plasma membranes by Glossman and Neville (1971) two points are immediately obvious. First, not all the surface-membrane glycoproteins contain fucose and, secondly, the Schiff reagent does not detect certain glycoproteins detected using the fucose radiolabel. It is also worthy of note that some of the fucose-containing glycoproteins reacted very weakly or not at all with Coomassie blue. It would consequently appear to be desirable, when analysing membrane-protein alterations in neoplasia, to employ 2 or 3 different methods of monitoring the protein components.

The lectin studies revealed alterations in several sugar moieties, particularly in the tumour nuclei (Table III). The apparent increases in the levels of galactose and sialic acid were the most marked. An increase in a chromatin-associated glycoprotein containing high levels of galactosamine in rat hepatoma has been reported (Yeoman *et al.*, 1976). Decreases in galactose and sialic acid in isolated nuclei, detected using fluorescein-labelled lectins, have also been described for rat hepatomas and sarcomas (Stoddart and Price, 1977). The nuclear fluorescence of the cells of the DMN-induced renal tumour apparently did not involve the nucleoplasm itself, but was restricted to the nuclear membrane. It may be that alterations of the glycosylated molecules of the nuclear membrane are a common feature of neoplasia.

Some of the saccharide alterations observed in the DMN-induced renal tumour may not have arisen solely as a result of malignant transformation. Kraemer (1971) has emphasized that altered sialic-acid levels in hepatoma may be due to "malignant dedifferentiation" of the organ, in addition to, or instead of, neoplastic transformation *per se*. This is a consequence of a cell-selection procedure. For example, 8 different cell types in normal rat kidney may become predominantly 2 or 3 cell types in the renal tumour. Any alteration in glycoprotein composition may consequently result from the enrichment of the cell "pool" in 1 or 2 types of cell. One method of overcoming this problem might be to maintain the kidney in tissue culture instead of in organ culture. Both normal and DMN-transformed rat kidney cells soon become mesenchymal under these conditions, and as the mesenchymal cells are the probable progenitors of the predominant tumour produced in rats, such an approach may allow meaningful comparative experiments to be performed.

We would like to thank Dr C. D. Ockleford for his assistance with the electron microscopy, and Dr R. W. Stoddart for permitting us to perform the lectin studies in his laboratory. Drs R. Borland and W. Jacobson were the sources of much advice and encouragement. During the course of this work A.W. was in receipt of a research scholarship from the Medical Research Council.

### REFERENCES

ALLISON, A. C. (1975) Antigens Shared by Tumour Cells and Foetal or Gonadal Cells. In *Immunobiology of Trophoblast* Ed. R. G. Edwards, C. W. S. Howe and M. H. Johnson. Cambridge University Press. p. 19.

AVRUCH, J. & WALLACH, D. F. H. (1971) Preparation and Properties of Plasma Membrane and Endoplasmic Reticulum Fragments from Isolated Rat Fat Cells. *Biochim. biophys. Acta*, **233**, 334.

BEEK, W. P. VAN, EMMELOT, P. & HOMBURG, C. (1977) Comparison of Cell-surface Glycoproteins of Rat Hepatomas and Embryonic Rat Liver. *Br. J. Cancer*, **36**, 157.

BENEDETTI, E. L. & EMMELOT, P. (1967) Studies on Plasma Membranes. IV. The Ultrastructural Localization and Content of Sialic Acid in Plasma Membranes Isolated from Rat Liver and Hepatoma. *J. Cell Sci.*, **2**, 499.

BOOTH, A. G. & KENNY, A. J. (1976) Proteins of the Kidney Microvillus Membrane. *Biochem. J.*, **159**, 395.

BORLAND, R. & HARD, G. C. (1974) Early Appearance of "Transformed" Cells from the Kidneys of Rats Treated with a "Single" Carcinogenic Dose of Dimethylnitrosamine (DMN) Detected by Culture *In vitro*. *Eur. J. Cancer*, **10**, 177.

BOSMANN, H. B., HAGOPIAN, A. & EYLAR, E. H. (1968) Cellular Membranes: the Isolation and Characterization of the Plasma and Smooth Membranes of HeLa Cells. *Archs Biochem. Biophys.*, **128**, 51.

BROCKAS, A. J. & WIERNIK, G. (1976) Isolation of Labelled Glycoproteins from Organ Cultured Carcinoma of the Cervix. In *Human tumours in Short Term Culture*. Ed. P. P. Dendy. London: Academic Press. 270.

BUCK, C. A., GLICK, M. C. & WARREN, L. (1970) A Comparative Study of Glycoproteins from the Surface of Control and Rous Sarcoma Virus Transformed Hamster Cells. *Biochemistry*, **9**, 4567.

COOK, G. M. W. & STODDART, R. W. (1973) *Surface Carbohydrates of the Eukaryotic Cell*. London: Academic Press.

DISCHE, Z. & SHETTLES, L. B. (1948) A Specific Color Reaction of Methylpentoses and a Spectrophotometric Micromethod for their Determination. *J. biol. Chem.*, **175**, 595.

FIALA, S. & FIALA, E. S. (1973) Activation by Chemical Carcinogens of γ-glutamyl Transpeptidase in Rat and Mouse Liver. *J. natn. Cancer Inst.*, **51**, 151.

FITZPATRICK, D. F., DAVENPORT, G. R., FORTE, L. & LONDON, E. J. (1969) Characterization of Plasma Membrane Proteins in Mammalian Kidney. I. Preparation of a Membrane Fraction and Separation of the Protein. *J. biol. Chem.*, **244** 3561.

GEORGE, S. G. & KENNY, A. J. (1973) Studies on the Enzymology of Purified Preparations of Brush Border from Rabbit Kidney. *Biochem. J.*, **134**, 43.

GLOSSMAN, H. & NEVILLE, D. M. (1971) Glycoproteins of Cell Surfaces. A Comparative Study of Three Different Cell Surfaces of the Rat. *J. biol. Chem.*, **246**, 6339.

HAMILTON, J. M. (1975) Renal Carcinogenesis. *Adv. Cancer Res.*, **22**, 1.

HARD, G. C. & BUTLER, W. H. (1970) Cellular Analysis of Renal Neoplasia: Induction of Renal Tumors in Dietary-conditioned Rats by Dimethylnitrosamine, with a Reappraisal of Morphological Characteristics. *Cancer Res.*, **30**, 2796.

HARD, G. C. & TOH, B. H. (1977) Immunofluorescent Characterization of Rat Kidney Tumors According to the Distribution of Actin as Revealed by Specific Antiactin Antibody. *Cancer Res.*, **37**, 1618.

HICKS, R. M., WALTERS, C. L., ELSEBAI, I., AASER, A., MERZABANI, M. & GOUGH, T. A. (1977) Demonstration of Nitrosamines in Human Urine: Preliminary Observations on a Possible Etiology for Bladder Cancer in Association with Chronic Urinary Tract Infections. *Proc. R. Soc. Med.*, **70**, 413.

HILL, M. J. & WILLIAMS, R. E. O. (1973) Interactions of Bacteria and Diet in the Etiology of Cancer. In *Host Environment Interactions in the Etiology of Cancer in Man*. Ed. R. Doll & I. Vogopija. Lyon: I.A.R.C. 231.

HULL, E., CARBONE, P., GITLIN, D., O'GARA, R. & KELLEY, M. (1969) Alpha-fetoprotein in Monkeys with Hepatoma. *J. natn. Cancer Inst.*, **42**, 1035.

KAADEN, O. R. & DIETZSCHOLD, B. (1974). Alterations of the Immunological Specificity of Plasma Membranes from Cells Infected with Marek's Disease and Turkey Herpes Viruses. *J. gen. Virol.*, **25**, 1.

KIMURA, A., NAGAI, Y. & TURUMI, K. I. (1961) Hexosamine and Sialic Acid Contents in Cells. *Nature, Lond.*, **191**, 596.

KRAEMER, P. M. (1971) Complex Carbohydrates of Animal Cells: Biochemistry and Physiology of the Cell Periphery. In *Biomembranes* Volume I. Ed. L. A. Manson. New York: Plenum Press. p. 67.

LOWRY, D. H., ROSEBROUGH, N. J., FARR, A. L. & RANDALL, R. J. (1951) Protein Measurement with the Folin Phenol Reagent. *J. biol. Chem.*, **193**, 265.

MAGEE, P. N. (1956) Toxic Liver Injury: the Metabolism of Dimethylnitrosamine. *Biochem. J.*, **64**, 676.

MAGEE, P. N. (1976) Some Illustrative Systems of Chemical Carcinogenesis: Nitrosamines. In *Scientific Foundations of Oncology*. Ed. T. Symington & R. L. Carter. London: Heinemann. p. 292.

MAGEE, P. N. & BARNES, J. M. (1956) The Production of Malignant Primary Hepatic Tumours in the Rat by Feeding Dimethylnitrosamine. *Br. J. Cancer*, **10**, 114.

NEVILLE, D. M. & GLOSSMAN, H. (1971) Plasma Membrane Protein Subunit Composition. A Comparative Study by Discontinous Electrophoresis in Sodium Dodecylsulfate. *J. biol. Chem.*, **246**, 6335.

OCKLEFORD, C. D. & WHYTE, A. (1977) Differentiated

Regions of Human Placental Cell Surface Associated with Exchange of Materials between Maternal and Foetal Blood: Coated Vesicles. *J. Cell Sci.*, **25**, 293.

PEARLSTEIN, E. & SEAVER, J. (1976) Non-lytic, non-ionic Detergent-extraction of Plasma Membrane Constituents from Normal and Transformed Fibroblasts. *Biochim. biophys. Acta*, **426**, 589.

RHOADES, J. W. & JOHNSON, D. E. (1972) N-dimethylnitrosamine in Tobacco Smoke Condensate. *Nature, Lond.*, **236**, 307.

ROBLIN, R., CHOU, I. & BLACK, P. H. (1975) Proteolytic Enzymes, Cell Surface Changes and Viral Transformation. *Adv. Cancer Res.*, **22**, 203.

STODDART, R. W. & KIERNAN, J. A. (1973) Aprotinin, a Carbohydrate-binding Protein. *Histochemie*, **34** 275.

STODDART, R. W. & PRICE, M. R. (1977) Membrane Saccharides of Rat Liver and Malignant-cell Nuclei. *Biochem. Soc. Trans.*, **5**, 121.

STONE, K. R., SMITH, R. E. & JOKLIK, W. K. (1974) Changes in Membrane Polypeptides that Occur when Chick Embryo Fibroblasts and NRK Cells are Transformed with Avian Sarcoma Viruses. *Virology*, **58**, 86.

SWANN, P. E. & MCLEAN, A. E. (1968) The Effect of Diet on the Toxic and Carcinogenic Action of Dimethylnitrosamine. *Biochem. J.*, **107**, 14.

WARREN, L. (1959). The Thiobarbituric Acid Assay of Sialic Acids. *J. biol. Chem.*, **234**, 1971.

WHYTE, A., LOKE, Y. W. & STODDART, R. W. (1978) Saccharide Distribution in Human Trophoblast Demonstrated using Fluorescein-labelled Lectins. *Histochem. J.*, **10**.

YEOMAN, L. C., JORDAN, J. J., BUSCH, R. K., TAYLOR, C. W., SAVAGE, H. E. & BUSCH, H. (1976) A Fetal Protein in Chromatin of Novikoff Hepatoma and Walker 256 Carcinosarcoma Tumors that is Absent from Normal and Regenerating Rat Liver. *Proc. natn. Acad. Sci. U.S.A.*, **73**, 3258.